# Collecting Early Childhood Obesity Measurements Through a Home Visiting Program: A Proof-of-Concept Study

**DOI:** 10.5888/pcd17.200214

**Published:** 2020-12-31

**Authors:** Julie M. Kapp, Brian Hall, Allison Kemner

**Affiliations:** 1 University of Missouri School of Medicine, Department of Health Management and Informatics, Columbia, Missouri; 2 Harry S. Truman School of Public Affairs, University of Missouri Columbia, Columbia, Missouri; 3 Parents as Teachers National Center, St. Louis, Missouri

## Abstract

Community-based home visiting programs are recommended vehicles for early life-course interventions to prevent childhood obesity. We developed and implemented a proof-of-concept protocol for collecting child weight and length or height data for children aged 6 months to 5 years through Parents as Teachers (PAT) affiliates that were geographically dispersed throughout the United States. We implemented our protocol with 1 affiliate in each of 4 states. We assessed formative measures of the implementation from parent educators and site leaders and reviewed delivery process measures. Findings suggest that collecting data on child measurements through an existing home visiting program is 1) feasible (91% of estimated measurements achieved); 2) does not require much time (median, 0.5 hours spent per child); 3) is a positive experience for families (71% of parent educators indicated that families enjoyed the experience); and 4) is fairly accurate (82% of collected data met eligibility and quality standards).

**Summary**

**What is known on this topic?**

Community-based home visiting programs are recommended vehicles for early life-course interventions to prevent childhood obesity.

**What is added by this report?**

We developed and implemented a proof-of-concept protocol for collecting measurements on child weight and recumbent length or standing height for children aged 6 months to 5 years in 4 states through a home visiting program.

**What are the implications for public health practice?**

The program is easily implemented, requires minimal time from program staff, and has positive engagement by parents. Opportunities include practical considerations of more equipment, ease of equipment portability, engaging hesitant children, and enhanced training.

## Introduction

Childhood obesity has a detrimental impact on physical and psychosocial (ie, emotional, social, and school functioning) health-related quality of life (1). Data from the National Health and Nutrition Examination Survey for 2011–2012 indicate that 7.1% of US children aged 0 to 24 months reached or exceeded the 97.7th percentile for weight-for-recumbent length (2). The World Health Organization recommends addressing obesity risk within a child’s first 1,000 days (conception to age 2 years) (3). Many risk and protective factors for childhood obesity, including a child’s dynamic eating patterns, responsive parenting, insufficient sleep, and a lack of physical activity appear within the first 1,000 days (4,5).

Community-based home visiting programs are recommended vehicles for interventions to prevent early childhood obesity (6), given their pragmatic approach facilitated by ongoing support for behavior change (7). The Parents as Teachers (PAT) home visiting program (parentsasteachers.org) is recognized federally as a national evidence-based home visiting model by the Department of Health and Human Services and Title IV-E Prevention Services Clearinghouse. Certified parent educators collect data through observations and parent self-reports, including screenings for developmental stages, depression, and intimate partner violence, through personal visits that occur primarily in the home. PAT provides parents with information on screening outcomes; helps parents understand health and wellness information, such as proper nutrition and the need for prenatal or child checkups; and helps connect families to resources for basic needs (eg, housing, education) to support a healthy family environment. Additionally, parent educators support parents with reflections and goal setting to encourage positive parenting behaviors and parent–child interactions. A family can enroll at any time from pregnancy through kindergarten, depending on the design or policies of the local affiliate and regardless of need or risk. A description of PAT affiliate performance, reach, and family diversity is available at https://tinyurl.com/yy5v5k3f (8).

## Purpose and Objectives

PAT has the potential to inform early childhood obesity prevention efforts with the addition of child measurements to their routinely collected data on the family and individual levels. Toward this end, we developed and implemented a proof-of-concept protocol for collecting child weight and recumbent length or standing height measurements for children aged 6 months to 5 years through local PAT affiliates that are dispersed throughout the United States.

## Intervention Approach

We used the Life Course Health Development framework (9) to inform this project. The framework views early childhood as a critical period sensitive to exposures and experiences and longitudinally monitors those influences in terms of health trajectories and health development.

Discussions, primarily with the Parents as Teachers National Center (PATNC) Vice President for Research and Quality (A.K.) and the PATNC Penelope Technical Specialist (B.H.), occurred at least twice per month. Our protocol development process included review of early childhood obesity literature; discussion of the value of collecting child measurements in the context of existing PAT data; discussion of the logistics of collecting child measurements; creation of an implementation strategy to invite sites and for child measurements; selection of inclusion and exclusion criteria for sites and children; review of the data and available sample; consideration for site readiness; diversity of service delivery area; review of child measurement standards; review of training materials (10–12), review of equipment; development of the protocol; and development of formative and process evaluation components.

The 11-page protocol shared with sites for collecting child measurement data included information on site eligibility, incentives, anticipated questions and answers, a timeline, contact information, written instructions and training for using the equipment (reinforced by a YouTube video, and in-person training by site leaders), eligibility criteria for the children, talking points for parent educators to ask participation of families, instructions and a checklist for parent educators when taking measurements (Appendix), instructions for data entry of measurements, the feedback process and instrument, and a commitment form.

To meet eligibility criteria, PAT affiliate sites were required to have implemented PAT’s evidence-based model, used PATNC’s data management system (compliant with the Health Insurance Portability and Accountability Act and nicknamed “Penelope”) for at least 12 months, met the PAT Blue Ribbon endorsement for data quality standards, and have a robust sample size of children (each with >100 eligible children). Among other criteria, eligible children had to be aged 6 months to 5 years as of September 1, 2019; to be still actively enrolled in PAT; and to have a completed child health record.

We invited leaders from 5 affiliates (representing a total of 1,078 children as of July 2019) that were diverse in their service delivery and location to participate in an informational webinar in September 2019. In the webinar, we shared the protocol, introduced the project, and answered questions. We asked affiliates to review the protocol over a week’s time and indicate through an online commitment form their willingness to participate. Four affiliates agreed; 1 declined because of the timing of leadership transitions. Locations were in South Carolina, Texas, Illinois, and Florida and included rural areas (eg, near Greenwood, South Carolina) and population-dense areas (eg, Fort Worth, Texas). Affiliates represented a range of geographic challenges in implementing their programs and reaching their families. For example, the South Carolina affiliate operates across 2 sites and 6 rural counties. The Illinois affiliate operates across sites that are 40 miles apart.

We shipped equipment (infant and toddler bath scales, infant measuring mats, tape measures, 9-volt batteries, and hand sanitizer) to the sites. PATNC provided sites with lists of eligible children. We provided incentives for meeting goals of 25 to 49 children ($100 Walmart gift card) or 50 or more children per site ($150 Walmart gift card). No incentives were provided to parents. Site leaders trained their parent educators and incorporated measurements into the program depending on the design or policies of the local affiliate. Some sites incorporated measurements into the next scheduled home visit or group connections designed to bring families and children together to share information about parenting issues and child health and development. Because more than 100 primary languages are spoken by enrolled families nationwide, sites may have included translation to languages other than English (affiliates often employ bilingual parent educators). Child measurement data collection ended in November 2019 and spanned approximately 6 weeks. Sites entered measurements directly into Penelope. We sent letters to families through PAT affiliates in spring 2020 to thank them for participating and to share project results.

## Evaluation Methods

We used a mixed-methods design to capture formative and process measures of the implementation. We asked participating parent educators to complete an anonymous 12-question Qualtrics survey (SAP AG) to provide quantitative and qualitative feedback. Questions included Why did you participate; What do you hope results from this effort; How many hours did you spend on the project; What went well; What were the challenges; What are your recommendations to solve those challenges; What are your impressions about how the parents felt about the measurements; How many children did you measure successfully (ie, both height/length and weight); How many children did you attempt to measure but could not; What are your thoughts on the protocol and training; What are your thoughts on the equipment; and an open-ended question for any other thoughts to share. Site leaders then participated in a close-out webinar to provide their perspectives on the process. The agenda, facilitated by PATNC personnel, included a question-by-question discussion of preliminary results of the parent educator survey. 

Finally, as a process measure of delivery, we assessed the percentage of data collected that met eligibility criteria or was biologically plausible. For a child younger than 24 months, weight-for-recumbent body length was compared with the World Health Organization sex-specific growth standards; for children 24 months or older, the CDC growth charts were used. We used CDC codes to calculate the percentiles and *z* scores for measurements of children aged 0 to less than 24 months (13) and 24 months or older (14). The code (13,14) flags extreme values and those that are biologically implausible; the websites provide detail and definitions. The University of Missouri institutional review board reviewed and approved this project.

## Results

### Parent educators

Of the 56 parent educators who participated in data collection, 38 (68%) completed a survey. Half of respondents (19 of 38, 50%) indicated they participated for practical reasons such as being asked or required to do so, and nearly half (18 of 38, 47%) participated out of interest or to be helpful to families. Predominantly, parent educators hoped this effort would help families develop awareness and that it would ultimately lead to better health outcomes. Parent educators reported spending a median of 2.9 hours on the project (range, 0 hours [reporting that measurements were incorporated into the home visit] to 22 hours) or a median of 0.5 hours per child that was successfully measured (range, 0–2.1 hours). The median number of children that parent educators attempted to measure but did not was 0 (range, 0–30 children). The predominant response to what went well was that families enjoyed it or were engaged, cooperative, or excited (27 of 38, 71%). Thirty-two educators (32 of 38, 84%) reported any challenges; predominant responses were uncooperative children (19 of 32, 59%) or challenges related to the equipment (7 of 32, 22%), such as it being cumbersome to carry or the ratio of scales provided to parent educators. Proposed solutions included distracting the children and providing more scales and bags. Most (27 of 38, 71%) parent educators felt the parents were positive about the experience (“happy and interested,” “pleased,” or “excited”); 4 respondents reported parental skepticism or that the parent felt it was the role of the doctor’s office. Regarding the protocol and training, 47% (18 of 38) had positive feedback (“easy,” “simple,” “clear,” “thorough”); 21% (8 of 38) had neutral feedback (“fine,” “okay”); and 21% (8 of 38) either did not receive the training from their sites or preferred more training. Regarding the equipment, 74% (28 of 38) said it was easy to use or had positive feedback; 8% (3 of 38) indicated it was bulky to carry; and 8% (3 of 38) had difficulty with the measurement units. Open-ended feedback included recommendations to provide results to parents about the project and handouts for the parents.

### Site leaders

All sites participated in the close-out webinar. Primary suggestions included providing more equipment to sites that experienced challenges with equipment-sharing due to their service delivery format, emphasizing in the training to be prepared to distract the children if they are uncooperative or hesitant, and providing a bag for the equipment. Other suggestions included decorating the equipment with stickers to make the process more fun for children and hands-on training for the parent educator using a doll for demonstration.

### Process measures

Sites completed 260 child measurements (Table), reaching 91% of the sites’ estimated goals. Overall, 17 children were measured for each set of equipment provided. Among the children measured, 214 (82%) met the eligibility and plausibility criteria.

**Table:** Proof-of-Concept Project Outputs for Measurements of Children Aged 6 Months to 5 Years as of November 2019, by State, Parents as Teachers Home Visiting Program, 4 US States

State	Estimated No. of Children Per Site	Actual No. of Children Per Site	Actual vs Estimated %	No. of Equipment Sets Sent to Site^a^	No. of Children Measured Per Set of Equipment	No. of Parent Educators	Parent Educator Survey Response Rate, %
Florida	125	87	70	3	29	11	45.5
Illinois	50	72	144	5	14	17	58.8
South Carolina	35	40	114	5	8	5	100.0
Texas	75	61	81	2	31	23	78.3
Overall	285	260	91	15	17	56	67.9

a Based on requests from the site.

## Implications for Public Health

Experts encourage child development initiatives to incorporate health behaviors rather than implement independent efforts around early childhood development, obesity prevention, and health promotion (5). Yet programs must consider pragmatic barriers before integrating additional prevention efforts into their workflow. Additional activities may infringe on or compete with a program’s existing model and commonly require additional funding and infrastructure (15), which is often not sustainable.

Positive findings from our project included favorable engagement by most families who participated and a limited time burden in taking measurements. Negative findings included uncooperative children and some difficulty with carrying the equipment and the measurement units.

Limitations include that we did not assess inter-rater reliability of the measurements. However, we would not expect minor variations to substantively affect weight-for-length percentile categories of overweight or obesity. Given selection criteria, the success of data collection from these sites may not represent the willingness, readiness, and capacity of all PAT sites. Strengths of this project include the robust sample size for a proof-of-concept study, the trilevel assessment (site leaders, parent educators, and process measures), and the geographic diversity of the sites. Future efforts will provide more equipment and bags to ease the burden of carry, use toys and stickers to engage hesitant children, and enhance the training and data collection form to further reduce measurement errors.

## Appendix Measurement Checklist From the Protocol for Measuring Children Aged 6 Months to 5 Years as a Proof of Concept, Parents as Teachers Home Visiting Program, 4 US States, 2019

- Sanitize the equipment before each child.
- Sanitize your hands before measurement.
- Be prepared with toys, etc, to distract the child during measurement if necessary.
- Take each measurement 2–3 times to ensure accuracy.
- Take the weight first.
- Recumbent weight (if the child is not standing well, generally before ages 12–15 months):
  - Make sure the scale is placed on a flat, hard surface (ie, the floor, not a carpet).
  - Make sure to zero the scale immediately before measuring. If you lay a towel on the scale, zero the scale with the towel on it.
  - Have the parent remove all clothing from the infant. If the infant needs to retain the diaper, ensure it is not wet or soiled because that will add additional weight.
  - Lay the child on his or her back in the tray. Ensure the child is not touching the tray or anything outside the tray.
  - Use the “Hold” button if the child is moving too much.
  - Measure 2–3 times to get an accurate assessment.
  - Immediately record the measurement to the nearest decimal (pounds and ounces).
- Standing weight (if the child is standing well, generally ages 12–15 months or older):
  - Remove the tray.
  - Make sure the scale is placed on a flat, hard surface (ie, the floor, not a carpet or rug).
  - Make sure to zero the scale immediately before measuring.
  - Have the child/parent remove the child’s shoes and heavy or bulky clothing.
  - Have the child stand up straight with their feet as indicated on the scale.
  - Measure 2–3 times to get an accurate assessment.
  - Immediately record the measurement to the nearest decimal (pounds and ounces).
- Recumbent length (children up to 24 months old; measure lying down, using the board):
  - Check that the child is not wearing shoes, bulky clothing, hair ornaments, or braids that may interfere with the measurement.
  - Follow the instructions provided with the equipment. They will probably read something like this:
    - Lay the child on the mat.
    - Ask the parent to assist by kneeling above the child’s head and holding his or her head flat against the headpiece, holding his or her head gently yet securely. A visible line from the child’s ear hole to the bottom of the eye socket should be perpendicular to the board or floor. Make sure the child’s chin is not tucked in or stretched too far back.
    - Ensure the child’s shoulders, back, and buttocks are flat against the board.
    - Place your left hand on the child’s knees to straighten the legs.
    - Place the movable piece against the child’s flat feet.
    - Check that the child’s position is correct, and note the measurement to the nearest 0.1 cm.
